# Guidance for statistical design and analysis of toxicological dose–response experiments, based on a comprehensive literature review

**DOI:** 10.1007/s00204-023-03561-w

**Published:** 2023-08-12

**Authors:** Franziska Kappenberg, Julia C. Duda, Leonie Schürmeyer, Onur Gül, Tim Brecklinghaus, Jan G. Hengstler, Kirsten Schorning, Jörg Rahnenführer

**Affiliations:** 1 Department of Statistics, TU Dortmund University, Vogelpothsweg 87, 44227 Dortmund, Germany; 2 Leibniz Research Centre for Working Environment and Human Factors at the Technical University of Dortmund (IfADo), Ardeystrasse 67, 44139 Dortmund, Germany

**Keywords:** Dose–response, Concentration–response, Alert concentration, Literature review

## Abstract

The analysis of dose–response, concentration–response, and time–response relationships is a central component of toxicological research. A major decision with respect to the statistical analysis is whether to consider only the actually measured concentrations or to assume an underlying (parametric) model that allows extrapolation. Recent research suggests the application of modelling approaches for various types of toxicological assays. However, there is a discrepancy between the state of the art in statistical methodological research and published analyses in the toxicological literature. The extent of this gap is quantified in this work using an extensive literature review that considered all dose–response analyses published in three major toxicological journals in 2021. The aspects of the review include biological considerations (type of assay and of exposure), statistical design considerations (number of measured conditions, design, and sample sizes), and statistical analysis considerations (display, analysis goal, statistical testing or modelling method, and alert concentration). Based on the results of this review and the critical assessment of three selected issues in the context of statistical research, concrete guidance for planning, execution, and analysis of dose–response studies from a statistical viewpoint is proposed.

## Introduction

In toxicological research, learning about the properties of compounds regarding their effects on humans, animals, or cells is one of the main goals. This often requires dose–response (or concentration–response, time–response, etc.) experiments, where multiple increasing doses of the compound of interest are administered to individual groups of experimental units. The analysis of these experiments can then be targeted at a comparison of the different doses against a negative control, or at the comparison of several treatments or several endpoint targets (e.g., genes) all receiving the same treatment, or at calculating alert concentrations. A central decision is whether to consider only the actually measured doses (e.g., in a display via barplots, with pairwise comparisons against the negative control), or whether an underlying (parametric) model is assumed and fitted to the data, allowing an interpolation between the measured doses.

When considering pairwise comparisons of the measured doses against a negative control, popular methods are given by the Dunnett test (Dunnett 1955), the Williams test (Williams 1971), and further extensions (Tamhane et al. 1996). In terms of alert concentrations, the lowest observed effective concentration (LOEC, Delignette-Muller et al. (2011)) or the no-observed adverse effect level (NOAEL, Dorato and Engelhardt (2005)) are potential approaches.

An alert concentration in general is defined as the concentration where the measured response value attains or exceeds a certain pre-specified threshold. This threshold can be based on absolute or relative values, and it may also contain significance statements (Kappenberg et al. 2021). Assuming an underlying model allows the calculation of alert concentrations that are not only restricted to the measured condition values, but also any value in between is possible. While the calculation of ED values (effective doses, Ritz et al. (2019)) is a frequent goal for viability assays, recently, the calculation of different model-based alert concentration has also been explicitly recommended for gene expression assays (Jiang 2013; Kappenberg et al. 2021; Möllenhoff et al. 2022).

Generally, the setup and analysis of a dose–response experiment consists of the three areas biological considerations, statistical design, and statistical analysis. In each area, before conducting or analysing the experiment, certain decisions need to be made. With respect to the first aspect, the biological considerations, based on the underlying toxicological question, the type of assay, and the corresponding type of exposure (e.g., dose, concentration, time) need to be specified. The second part concerns the (statistical) design of the experiment. Several decisions, also supported by statistics, have to be made here, such as the number of considered doses, the actual dose values, and the respective sample sizes. As the third step, statistical analyses are performed. These include the display of the results, the choice of aspects to be analysed, the test or modelling method to be used, and, if applicable, the alert concentration to be calculated. Also, regarding statistical analyses, decisions about the appropriate approaches need to be made.

Hothorn (2014), Hothorn (2016) give an overview over the statistical considerations when analysing dose–response experiments. This includes the aspects about the display of the data, the discussion about how to report results of data analyses (*p*-values versus confidence intervals), an overview of tests for specific scenarios, and modelling considerations. A comparison of different model-based alert concentrations is given in Jensen et al. (2019).

However, there is a discrepancy between the state of the art in statistical methodological research and published analyses in the toxicological literature. To provide targeted recommendations for potential improvements of the published analyses, there is a need to quantify the extent of this discrepancy with respect to the different aspects of dose–response experiments. Thus, in this paper, an extensive literature review in three major toxicological journals (‘Archives of Toxicology’, ‘Cell Biology and Toxicology’, and ‘Toxicological Sciences’) is presented. All publications from 2021 were screened for dose–response analyses, and the identified analyses were evaluated. The review was performed in terms of the relevant questions about the decisions in the three areas biological considerations, statistical design, and statistical analysis. Specific aspects as described above represent the relevant questions, the answers to which provide a comprehensive picture of the current state of the art in the published literature.

The remainder of this paper is structured as follows. First, the literature review and how it was conducted is presented in detail. All variables collected and the possible expressions are explained in context. Then, the results of the literature review are presented, both by extensive univariate analyses of all variables, and by some bivariate considerations. Selected results of the literature review are critically discussed and placed in the state of the art of statistical research. Based on the results from the literature review, concrete guidance is offered for planning, executing, and analysing a dose–response experiment from a statistical point of view.

Throughout the paper, the analyses are often referred to as ‘dose-response analyses’, and the alerts are often referred to as ‘alert concentrations’. From a statistical viewpoint, the different types of exposures are equivalent. Therefore, whenever it is not explicitly specified, ‘dose-response’ or ‘alert concentration’ also refers to all other types of exposures, respectively. Additionally, in this work, the word ‘condition’ is used as a general term. This term includes all types of exposures such as concentrations, doses, times, frequencies, and intensities.

## Literature review

For the literature review, three high-ranking journals with a rather broad scope and a clear relevance to toxicological research were chosen. The three journals are ‘Archives of Toxicology’, ‘Cell Biology and Toxicology’, and ‘Toxicological Sciences’. All publications from 2021 from these three journals were considered and screened for dose–response (concentration–response, time–response, etc.) analyses. Only published figures were considered in this screening, and not dose–response analyses that are summarized only in tables or presented only in supplemental figures. An analysis was only included in the review when at least three conditions plus a negative control, or at least four conditions if no control was available, were shown. Differential equation-based plots [e.g., physiologically based pharmacokinetic (PBPK) modelling] were not considered. If a figure displayed several dose–response analyses, e.g., different compounds or the effect of one compound on different biomarkers, each analysis was considered individually.

The review was performed by several reviewers. To unify the results, a comprehensive catalogue of variables was prepared in advance, together with their respective possible expressions and some additional explanations and remarks. The potential problem of inter-reviewer variability was addressed by holding frequent meetings and discussions, as well as extensive sample checks and unifying efforts.

### Considered variables

In this section, the variables collected and the possible values they can take are explained in detail. Additionally, a summarized overview over these variables, their possible values, and some comments is presented in Table 1.

**Table 1:** Overview of all variables considered in the literature review, together with possible values they can take and further details and comments as appropriate

Variables	Possible values	Comments
**Biology**		
Type of assay	− Viability − Enzyme activity − In Vivo − Proliferation − Mutagenicity − Gene expression − Protein − Other	
Type of exposure	− Concentration or Dose − Time − Frequency or Intensity − Other	
**Statistical design**		
Number of conditions	Number of conditions (e.g., doses or time points) displayed, without counting the control	Information whether a control is available was also collected
Design	Design information was obtained from author statements and own evaluations of measured condition values	
Sample size	Number of replicates for the control and for the non-control conditions, counted separately	
**Statistical analysis**		
Type of display	− Barplot − Curve-Interpolated − Scatter − Boxplot − Curve-Modelled	In addition, it was evaluated if significance was assessed via stars or via a compact letter display
Analysis goal	− Comparison-Cond0 − Comparison-Treat − Comparison-Tukey − AlertConc − Entire Curve	Multiple goals per analysis were possible. The ‘comparison’ goals always refer to some kind of statistical testing
Testing method	Statistical testing method(s) that were used	Global pre-tests and post hoc tests were both considered, if applicable
Modelling method	Method for modelling the curve, including linear and non-linear interpolation, as well as different parametric models	
Alert concentration	− ED value − BMD − NOAEL − ALEC	
Software	Software used for data analysis	

The variables can be categorized into groups, depending on the subject area (biology or statistics) and the stage (design or analysis) of the experiment. The two variables considering the type of assay and the type of exposure are biological considerations in a dose–response experiment, motivated by the toxicological research question. The number of conditions, the overall design, and the sample sizes belong to the statistical area of design of experiments, where the overall goal of the analysis must also be considered when decisions are made. Finally, the types of display, the analysis goal with regard to the decision for an analysis strategy, the testing method, the modelling method, and the alert concentration are statistical analysis considerations.

#### Type of assay

The type of assay can take one of the categories ‘Viability’, ‘Enzyme Activity’, ‘In Vivo’, ‘Proliferation’, ‘Mutagenicity’, ‘Gene Expression’, ‘Protein’, and ‘Other’. Some assays based on enzymes (e.g., the LDH assay) are used for indication of cytotoxicity, so they would fit in both categories ‘Viability’ and ‘Enzyme Activity’. Here, a distinction was made, whether the assay considers only one enzyme, in which case it was assigned to the ‘Enzyme Activity’ category, or several enzymes, in which case it was assigned to the ‘Viability’ category. In cases, where, e.g., protein or gene expression measurements from cells after an in vivo treatment (e.g., giving a compound to rats or mice and then harvesting their cells after sacrificing them) were considered, they were assigned to the respective category ‘Protein’ or ‘Gene Expression’, but not to the category ‘In Vivo’. The ‘In Vivo’ category only refers to measurements directly taken from the animals, e.g., body weight, heights, or diameters of organs.

#### Type of exposure

The types of exposure are divided into the four categories ‘Concentration or Dose’, ‘Time’, ‘Frequency or Intensity’, and ‘Other’. Typically, ‘Dose’ refers to the total amount of a compound that is administered (to, e.g., tissue or animals), while ‘Concentration’ describes the amount of a compound in a mixture that is applied to, e.g., cells. Regarding the ‘Time’ category, no difference was made between the exposure time and the time between the administration of a treatment and the measurement of the outcome variable. In terms of statistical modelling, the different types of exposures are considered to be equivalent.

#### Number of conditions

The number of conditions were counted, where the control itself did not count. However, it was additionally recorded, whether a control was present. Here, it has to be noted that for some dose–response analyses, some normalization with respect to the controls is typically conducted, and the control is not displayed in each case. In the review conducted here, this was considered as if no control was present, since only the data actually shown in the figures were considered.

For some figures, e.g., with the display on logarithmic axes, the exact number of conditions could not or not exactly be retrieved. In cases where some reasonable range for the number of conditions could be determined, spanning maximally three numbers (e.g., ‘6 to 8’), for the analysis, the smallest respective value was selected (i.e., 6 in this example).

#### Design

For the design, several aspects were considered. First, it was checked whether the authors of the respective paper stated anything specific about the choice of design of the experiment. In a second step, wherever possible, the actually measured condition values were retrieved from the data. Typical designs in toxicology are additive (‘equidistant’) designs and multiplicative (‘log-equidistant’) designs in which, starting from some initial condition value, the remaining condition values are obtained by adding a fixed value or multiplying with a fixed value.

To assess the retrieved values in terms of equidistance or log-equidistance, successive differences and ratios of the condition values, excluding the control, were calculated. These successive differences and ratios were then assessed with respect to their *arithmetic complexity*, i.e., the number of different values occurring in these successive differences. An arithmetic complexity of 1 for the successive differences thus corresponds to an equidistant design; an arithmetic complexity of 1 for the successive ratios corresponds to a log-equidistant design.

A dose–response analysis consisting of only three condition values (and a negative control) always leads to arithmetic complexities of at most two for both the differences and the ratios. Thus, the analyses with three condition values that did not follow an exactly equidistant or exactly log-equidistant design (i.e., that did not have an arithmetic complexity of 1) were counted separately in the category ‘unstructured 3 conditions’. All analyses that resulted in an arithmetic complexity of 2 for the successive differences and a value higher than 2 for the successive ratios were assumed to be ‘almost equidistant’, and all analyses in which this relationship was reversed were assumed to be ‘almost log-equidistant’.

The remaining dose–response analyses were considered to follow a different or more complex design and were analysed in more detail.

#### Sample size

The sample sizes, i.e., the number of replicates per condition, were collected separately for the control and the non-control conditions. Especially, for the non-control conditions, sample sizes can vary between conditions, or are stated as ranges across several experiments. In the case where ranges did not extend over more than four numbers (e.g., ‘3 to 6’ replicates), for the analysis, the value was set to the respective smallest value (i.e. 3 in this example). Some ranges that spanned more than four numbers but were within the range of 1 to 10 were summarized into an own category, and so were all other ranges that did not fit the above mentioned summarizing criteria.

#### Type of display

For the types of display, five different categories were considered. The first category is given by barplots, where no differentiation was made between pure barplots and such barplots that additionally provided information about standard errors or standard deviations. The next category is called ‘Curve-Interpolated’. Here, both linear and non-linear interpolation, i.e., the joining of dose-wise mean response values, were considered. The category ‘Scatter’ describes situations where only the data points were shown. In the category ‘Boxplots’, both boxplots alone and boxplots with superimposed individual data points were considered. The final category is called ‘Curve-Modelled’, where an underlying parametric or non-parametric model was assumed and fitted to the data.

In addition to these five categories, it was evaluated whether some significance statement was included in the figure. Here, it was distinguished between stars and between the so-called ‘(compact) letter display’ (CLD) (Piepho 2004). Stars indicate significant differences between the response values for a specific condition value and the response values for the control or another clearly specified condition value. The CLD assigns letters to treatment groups in such a way that if two or more groups share the same letter, no significant difference can be found between them. The CLD thus requires significance statements for all pairwise comparisons between the considered doses, while stars are typically provided for individual comparisons against the (negative) control.

#### Analysis goal

The next variable is the analysis goal, which was identified from the figure and the corresponding caption. It was possible to identify several analysis goals for one dose–response analysis. The possible goals include ‘Comparison-Cond0’, i.e., a comparison against the lowest tested condition value (often the control), ‘Comparison-Treat’, i.e., a comparison between different dose levels / treatments, or between different biomarkers all treated with the same compounds, and ‘Comparison-Tukey’, i.e., all pairwise comparisons between doses. These comparisons all require some kind of statistical testing. In addition, possible analysis goals were ‘AlertConc’, i.e., the explicit calculation of an alert concentration (see also the variable about alert concentrations), and the category ‘Entire Curve’, which refers to general observations about the shape of the dose–response analysis. No actual dose–response modelling was required for assignments within this category.

#### Testing method

For evaluation of the testing method, information about a global testing procedure (e.g., analysis of variance) and about a local testing procedure (e.g., pairwise comparison with the negative control) was collected. The testing method was identified as described in the corresponding paper and later categorized into the groups ‘ANOVA / Kruskal-Wallis / Friedman (only)’ (i.e., only a global test was performed), ‘t-test / multiple comparison / Bonferroni’, ‘t-test or ANOVA’, ‘Dunn / Dunnett / Steel / Sidak’, ‘Holm / Holm-Sidak’, ‘Least Significant Differences’, ‘Mann Whitney U / Wilcoxon’, ‘Tukey / Tukey-Kramer’, and ‘Duncan / Newman-Keuls’. For the local testing procedures, it was additionally evaluated whether the local tests were preceded by a global test, i.e., whether the overall testing procedure was performed in a two-step way.

ANOVA here refers to the analysis of variance, a statistical method to test for global differences between means of response values for different groups, where the response is assumed to follow a normal distribution (Chambers and Tibshirani 1992, e.g.). The Kruskal–Wallis test is a non-parametric, rank-based alternative for the same analysis goal (Kruskal and Wallis 1952), and the Friedman test is another non-parametric alternative, for which paired samples are assumed (Friedman 1937).

Student’s *t*-test is used for comparing the means of two groups while assuming a normal distributed response. The Bonferroni method is a very simple commonly used method for adjusting *p*-values from multiple tests to avoid an inflation of the type I error and to control the familywise error rate (FWER) (Bonferroni 1936).

The Dunnett procedure is used for multiple comparisons against, in this context, a (negative) control, by taking the correlation between the comparisons into account and thus increasing the power of the overall testing procedure while still controlling the FWER (Dunnett 1955). It is a parametric procedure. The Dunn procedure, which is also parametric, has a similar goal, but it is not restricted to testing against a negative control, but testing for differences between any pre-defined subset of all possible pairs of conditions (Dunn 1961). Steel’s test is a non-parametric procedure for simultaneously comparing different conditions against a negative control (Steel 1959). The Šidák (or Dunn–Šidák) test procedure is a method to control the FWER for independent tests using a modified significance level $\alpha$ (Šidák 1967).

The Holm (step-down) procedure is a more powerful alternative to the Bonferroni procedure, also controlling the FWER, but generally leading to fewer type II errors by pursuing a sequential strategy (Holm 1979). The Holm–Šidák method is a modification of the Holm method, also proposed by Holm (1979), where the respective critical value is calculated in a different way, also pursuing the goal of increasing the power of the procedure while controlling the FWER.

The least significant differences procedure works by calculating the smallest difference for which a comparison of means in the specific scenario is significant, based on normal distribution assumptions. All actually observed differences with a larger value than the initially determined smallest value then correspond to rejections of the respective null hypotheses (Fisher 1935).

The Mann–Whitney U test and the Wilcoxon test are two equivalent tests for comparing the means of two groups based on ranks, i.e., with a non-parametric procedure (Mann and Whitney 1947; Wilcoxon 1945).

The Tukey test is an approach for simultaneously determining the significance of all pairwise comparisons. Similar to the simple *t*-test, standardized differences of means are compared to some quantile, but the quantiles stem from the studentized range distribution and thereby the procedure controls the FWER (Tukey 1949). The Tukey–Kramer procedure is a modification of the Tukey procedure that also allows unbalanced sample sizes in the groups (Kramer 1956, 1957).

The Newman–Keuls method is a multiple comparison procedure similar to the Tukey procedure. However, it aims at having a higher power by choosing different critical values, which may result in not keeping the specified level $\alpha$ (Newman 1939; Keuls 1952). Duncan’s new multiple range test is a further modification of the Newman–Keuls method, aiming at an even higher power (Duncan 1955).

#### Modelling method

The variable modelling method captures both interpolation-based modelling and parametric or non-parametric modelling of a curve. The category ‘Linear Interpolation’ describes the joining of mean response values of the different doses via a piecewise linear function (Encyclopedia of Mathematics, a). ‘Nonlinear Interpolation’ also refers to a joining of the dose-wise mean response values, however here, not via a linear function, but via some non-linear function, e.g., via splines (Encyclopedia of Mathematics, b).

In terms of parametric models, the first category is the ‘Log-logistic/Hill/(sig)Emax’ model. All three names refer to equivalent parameterizations of the same family of models. The curve has a monotonous sigmoidal shape with, depending on the choice of the number of parameters, flexibly estimated values for the asymptotes, the inflection point, and the slope (Ritz et al. 2019; Ritz 2010; Fang and Zhou 2023). The category ‘Nonlinear Curve’ refers to all modelled curves for which the exact model name was not given. In comparison to the log-logistic model, for the ‘Exponential model’, the curve does not become saturated for high doses, but it always increases in an exponential way (Bretz et al. 2005). The category ‘Linear Model’ refers to the classical linear regression approach, and ‘Model selection’ means that several models were considered, and one model was chosen according to some criterion.

In contrast to the multiple comparison procedures, where the dose values are considered as qualitative (sometimes ordinal) observations, the condition values are always considered on a quantitative scale in modelling approaches. Modelling allows both interpolation between and extrapolation beyond the actually measured condition values.

#### Alert concentration

For analyses with some kind of dose–response modelling, the considered alert concentrations were grouped into four categories: The first category, ‘ED-Value’, considered effective doses/concentrations. An effective dose is defined as the dose for which a pre-specified (relative) effect can be observed in the response variable, e.g., the ED10 refers to the dose where 10% of the overall effect can be seen (Ritz et al. 2019; Sebaugh 2011, e.g.). This category also includes inhibitory concentrations, which are defined analogously, but refer to the inhibition of a biological process by a compound (Sebaugh 2011). The benchmark dose (BMD) approach gives an estimate of the ‘point of departure’, i.e., the lowest dose for which a response different from the background risk is observed (Jensen et al. 2019). ‘NOAEL’ describes the no-observed adverse effect level, also sometimes referred to as the ‘NOEC’ (no-observed effect concentration), which is the highest concentration for which no significant or relevant effect in comparison to the control can be seen (Dorato and Engelhardt 2005; Delignette-Muller et al. 2011). The ‘ALEC’ (absolute lowest effective concentration) is a model-based alert and is calculated as the concentration where the modelled curve intersects with a pre-specified effect level (Jiang 2013; Kappenberg et al. 2021).

#### Software

It was assessed which statistical software was used for the data analysis. This was only possible if explicitly stated by the authors, and was evaluated purely per paper, i.e., for each paper, only one software (or a combination of several softwares) was considered.

### Analysis strategy

Barplots are generally used to display the results of the literature review. For several variables, two presentations of the results are shown, one in which each dose–response analysis was considered individually, and one in which results were analysed on a per-paper basis. This per-paper approach means that for a variable, e.g., ‘Type of Exposure’, each possible value, in this example Conc/Dose, Time, Frequency/Intensity, and Other, was counted only once per paper. Therefore, if, in one paper, three curves with concentration as exposure, and ten curves with time as exposure were considered, both Conc/Dose and Time were counted once only for that particular paper. In the approach where each analysis was considered individually, the three and ten curves counted three and ten times, respectively. The per-paper approach helps avoiding structural bias in the results, when for example one paper considered an unusually high number of analyses of the same type of condition, which would result in a very high increase of observations for that specific type, but this would not necessarily reflect what is done typically in publications. However, this per-paper analysis was not conducted for all variables, since many of them (e.g., analysis goals, used statistical methods) are not based on decisions that are made solely by experimenters, but based on the goals and general situations of the different experiments.

## Results of the literature review

In this section, the results from the literature review are presented. First, univariate analyses for each variable are presented, followed by some selected bivariate relationships. Finally, the main results of the analysis are critically evaluated and discussed.

The analysis of the literature review was conducted in the statistical programming software R, version 4.2.2 (R Core Team 2022). For the graphical display of results, the package ggplot2 (Wickham 2016) was used, with the additional packages ggmosaic (Jeppson et al. 2021) and gridExtra (Auguie 2017).

### General situation

**Fig. 1:**
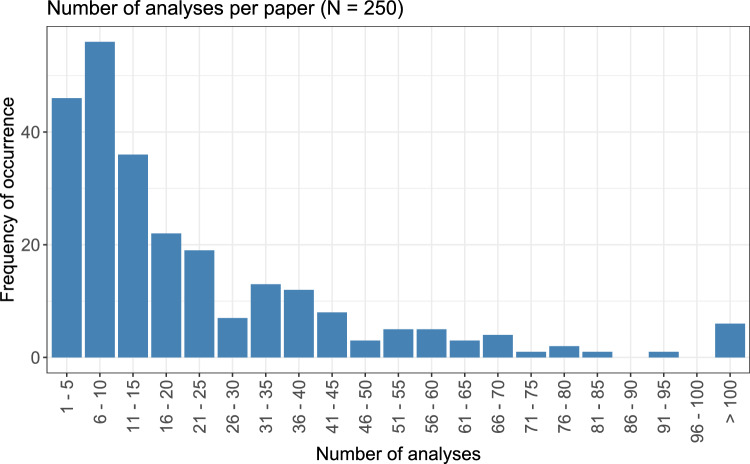
Number of considered dose–response analyses per paper, summarized to groups of five numbers, respectively. All numbers higher than 100 were summarized to the same category

In the three considered journals, a total of 1644 papers were published in 2021. Out of these 1644 papers, 250 contained at least one dose–response analysis fitting the selection criteria. In total, 5670 dose–response analyses were considered for the review. The number of dose–response analyses per paper is summarized in Fig. 1. While for the majority of papers, 1–15 dose–response analyses were performed, with a peak in the plot at 6–10 analyses, there exist some papers with a higher number of analyses: The range between 30 and 45 analyses was still frequently observed, and only slightly lower numbers could be found for the range from 50 to 70. Some papers even contained more than 100 analyses, these were summarized to one category in the displayed plot. The average (arithmetic mean) of considered analyses per paper is 22.7, the median is 14, and the quartiles are 6 and 31.

### Type of assay

**Fig. 2:**
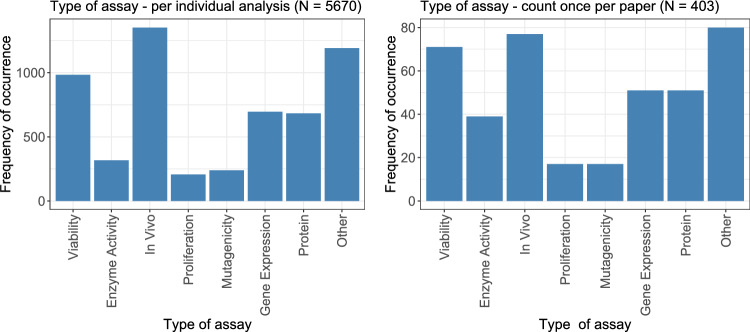
The types of assays considered in the dose–response analyses were counted. On the left, the display is given per individual analysis; on the right, each assay was counted only once per paper

First, the type of assay is evaluated. Figure 2 shows the frequency of occurrence for each type of assay, both on a per-analysis level (left) and on a per-paper level (right), with each assay counted only once per paper. This per-paper approach yields 403 observations. Both distributions are similar: Not considering those analyses that are categorized as ‘Other’, the class of ‘In Vivo’ assays was observed most often, followed by viability assays. ‘Gene expression’ and ‘Protein’ assays occurred similarly often, with still relatively high frequency, followed by the ‘enzyme activity’ assay. Finally, ‘proliferation’ and ‘mutagenicity’ assays were the ones with the lowest frequency.

### Type of exposure

**Fig. 3:**
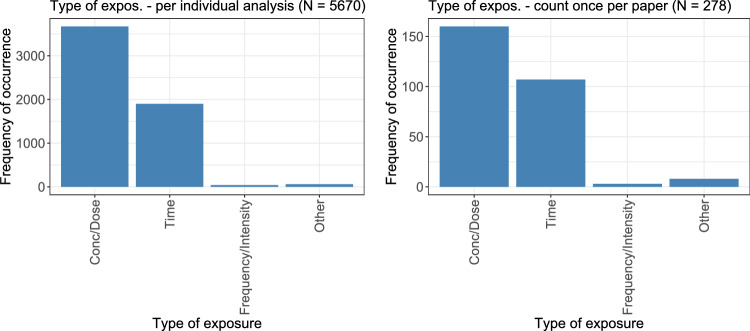
The types of exposures considered in the analyses are counted. On the left, the display is given per individual analysis; on the right, each exposure was only counted once per paper

The numbers of occurrences for each type of exposure are shown in Fig. 3, both on an individual level (left) and summarized per paper (right). On a per-paper level, 278 observations were made. With 250 papers considered in total, this means that, in the vast majority of papers, only one type of exposure according these four categories was considered. Dose–response or concentration–response analyses were the most common ones, followed by time–response analyses. Frequencies, intensities, and other exposures occurred only rarely.

### Number of conditions

**Fig. 4:**
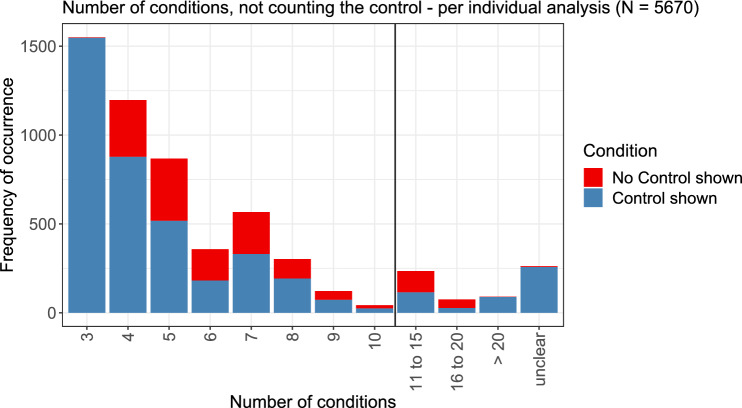
Number of conditions considered for each dose–response analysis, not counting the control. Those analyses for which a control was additionally shown are displayed in blue, the others are displayed in red. Numbers larger than ten were summarized in three categories (color figure online)

The numbers of conditions per analysis, not counting the control, are displayed in Fig. 4. The blue parts of the barplots indicate analyses for which a control was shown in addition to the stated number of conditions, and the red parts indicate analyses for which no control was shown. Due to the inclusion criteria for the literature review, no analyses with fewer than three conditions and additional control or fewer than four conditions without control are shown in this plot.

Measuring and displaying a control was more common than the lack of a control. Still, no control was shown for 1431 of the considered analyses. Three conditions was the most frequent case, followed by four and five conditions. Higher numbers were observed only in a few cases, but also more than 10 or even 20 conditions were sometimes observed. For some analyses, e.g., due to logarithmic axes that were difficult to read or due to overlapping dose–response analyses displayed in the same figure, no number could be retrieved.

Three doses in addition to a negative control is a very typical design, consisting of a ‘low’, a ‘medium’ and a ‘high’ dose (Hothorn 2016). This was also the minimal number of conditions for the respective dose–response analyses to be included in this review. Whenever a modelling approach is used for the analysis of the dose–response data, more conditions generally lead to better model fits due to more available information, but at least as many conditions (including the controls) as parameters in the fitted model are required to ensure identifiability.

### Design

The first step regarding the analysis of the designs that were used was to find out whether the authors of the papers explicitly referred to some statistical consideration about the chosen design. However, such information was not found in any of the papers. Thus, as a second step, wherever this was possible, the actual condition values were retrieved and the arithmetic complexity of resulting successive differences and ratios, as explained above, was used to obtain information about the underlying design.

**Fig. 5:**
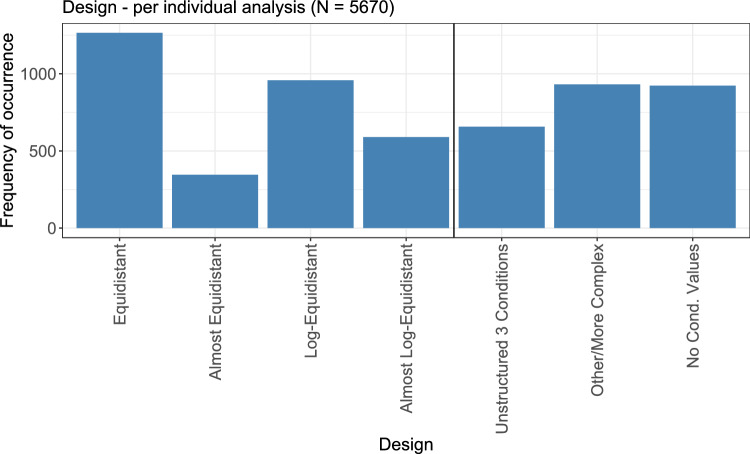
Analysis of the different designs for the condition values as they result from the calculation of the arithmetic complexities of successive differences and ratios of the actual condition values

The condition values could be retrieved for 4747 out of all 5670 analyses. For 36 analyses, the successive ratios were not calculated, due to the zeros in the corresponding series of condition values $-28, 0, 0.92, 2, 3, 28$ (no obvious design, 3 occurrences) and $-3, 0, 3, 6, 9$ (equidistant design, 33 occurrences). Figure 5 shows the results for the different designs, as obtained from an analysis of the arithmetic complexities.

Out of all categories, an equidistant design (i.e., additive condition values) occurred most often, followed by a log-equidistant (i.e., multiplicative) design. When also considering almost equidistant and almost log-equidistant designs, the two types additive and multiplicative were observed similarly often.

A class of designs in the category ‘other/more complex’ are designs that are in principle multiplicative (log-equidistant), but have one additional condition value in between that does not fit to the log-equidistance profile. One such example are condition values 0.1, 1, 5, 10, with three log-equidistant values and one additional condition value in between. Generally, many of the analyses in the ‘other/more complex’ category visually seem to be close to equidistant or log-equidistant designs, but with more than one additive or multiplicative factor.

The role of the negative control, especially in modelling approaches, is to estimate the left-sided asymptote of the response values. Similarly, a very high value could be added to the design to approximate a value of ‘infinity’, and thus help estimating the right-sided asymptote of a fitted model. Based on the retrieved condition values from the reviewed papers, an unusually extreme value for the highest condition was only observed in one paper, where the time in hours was measured for the time points 0.1, 1, 2, 3, 4, 5, 6, 20. However, the authors did not give specific reasoning for this, and the response values were displayed via barplots and not via a modelled curve, such that in all considered publications very high dose levels were never used for the improved estimation of the right-sided asymptote.

More details on the properties and advantages of suitable statistical designs are given in Section “Evaluation”.

### Sample size (number of replicates)

**Fig. 6:**
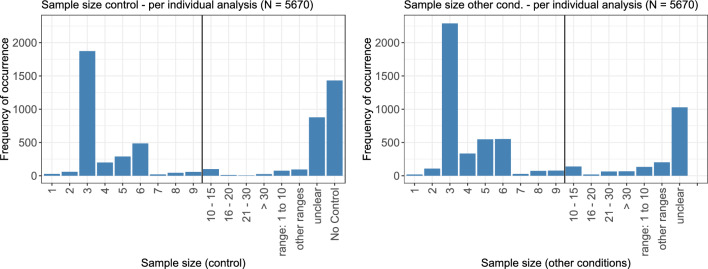
Sample sizes per condition for the control (left) and the other conditions (right). Small ranges (e.g., 2–3) were summarized to the respective smaller value (2 in this example), other ranges were either assigned to the category ‘range: 1 to 10’ or ‘other ranges’, depending on the values

For the sample size, the control (if available) and the non-control conditions were considered separately. Figure 6 shows the number of replicates per concentration for the control (left) and the non-control conditions (right). If applicable, ranges were summarized as explained in Section “Analysis strategy”. In both plots, the by far largest peak can be seen for the common number of three replicates per condition. Six, five, and four replicates follow in that order. Only very few analyses used less than three or more than six replicates. For a notable number of analyses, no exact information about the sample sizes could be found in the respective publication.

A very small sample size, due to ethical or cost-related reasons, is one of the main challenges of toxicological data (Hothorn 2016). In general, it is recommended that the sample size for the negative control should be a certain factor higher than the sample size for the other conditions. One possible choice for this factor is the square root of the number of conditions. Reducing the sample size as much as possible, while keeping the power of statistical testing at a reasonable level, has been a relevant topic in research for a long time (Schütz and Fuchs 1982), and often goes hand in hand with the optimization of the statistical design of experiments. Research is also conducted in the direction of incorporating historical control data into new experiments, to increase the sample size for the negative control (Kluxen et al. 2021; Hayashi et al. 2011).

When directly comparing the sample sizes in the literature review, for 3259 out of the 5670 analyses, the sample sizes for the control and the other conditions were the same or stated to be at least in the same range. For 804 analyses, no information was given for either the control or the other conditions, and for 1431 analyses, no control was shown. Only for 66 analyses, the control had a larger sample size than the other conditions, and for 18 analyses, it was the other way around. For the remaining analyses, no comparison could be performed due to missing values for either the control or the other conditions. For modelling via the commonly used four-parametric log-logistic model, Wang and Yang (2014) proved that the locally *D*-optimal design, i.e., the design minimizing the simultaneous confidence region for all four parameters, consists of four different support points. Li and Majumdar (2008) derived that one of these support points corresponds to the condition value 0. The sample size should be equal at all four support points, such that overall 25% of all samples should be allocated to the control (Silvey 1980). Although only few of the reviewed papers actually fitted a log-logistic model to the dose–response relationship, in general, the sample size in the control seems to be rather too low.

One challenge that has not been directly addressed here is the different approaches to dealing with technical and biological replicates. Sometimes, a first processing step is to calculate the mean values of all technical replicates per dose and then proceed, making use only of the biological replicates for estimation of the variability per dose. However, this leads to a loss of information and should thus be avoided (Ritz et al. 2019). Another challenge not addressed here, but nevertheless important, is the consideration of batch effects that can occur when combining data. These can be of biological nature, such as inter-donor differences in primary human cells, but also of technical origin, such as the use of different lots of a test substance. Possible influences on the variability of the data should be known on the experimental side to avoid or minimize them as well as on the statistical side to be able to take them into account in the statistical analysis.

### Display

**Fig. 7:**
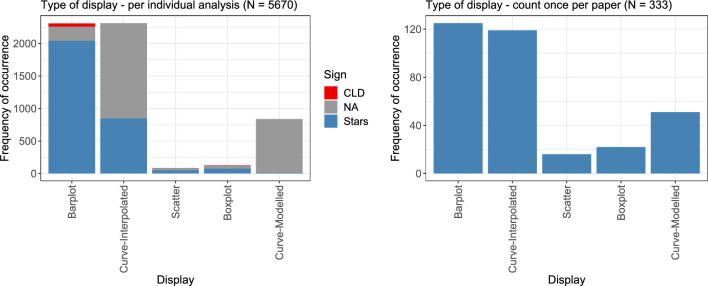
Number of times each type of display was used for the presentation of dose–response analyses, both on a per-analysis level (left), and when considering each display only once per paper (right). For the individual display, it is additionally indicated whether statements of significance were made in the figure, either via stars (blue) or via the CLD (red) (color figure online)

For the display of the respective dose–response analysis, both the overall type of display, and, if applicable, the display of significance were assessed. Overall, for 3076 of all 5670 analyses, information about the significance was given, in the vast majority of cases using stars. Figure 7 summarizes the type of display, once per individual analysis with additional information on significance statements (left), and once with counts per paper only, resulting in 333 observations (right).

Barplots and interpolated curves, i.e., mostly linearly joined data points (see Section “Modelling methods” for more details on the interpolation methods), clearly dominate the chosen displays. Barplots were almost always combined with some significance statements, almost exclusively making use of stars, but rarely also by making use of the compact letter display (CLD). Generally, a mean response value is displayed by barplots, often with error bars indicating standard deviations or standard errors.

Boxplots display the median, the two quartiles and, depending on the specific choice of the whiskers, the range of the data and the extreme values (‘outliers’). Although they generally convey far more information than barplots, boxplots were only very rarely used, even though some toxicological papers explicitly recommend using boxplots (Elmore and Peddada 2009; Pallmann and Hothorn 2016). Barplots and boxplots share the problem that the relative differences between the actual values of the variable on the x-axis are not displayed and thus do not allow an intuitive assessment of the overall dose–response relationship. For very small sample sizes, boxplots are hard to interpret. However, boxplots still comprise far more information than barplots which also implicitly assume normally distributed data (Hothorn 2016; Pallmann and Hothorn 2016).

For modelled curves (i.e., with underlying models, mostly parametric models), basically never was significance information provided. Significance typically refers only to the actually measured doses, whereas parametric modelling additionally allows interpolation between the doses.

When counting each display only once per paper (Fig. 7, right plot), the proportion of boxplots was larger, but, generally, the results are very similar to the individual analysis.

### Analysis goal

**Fig. 8:**
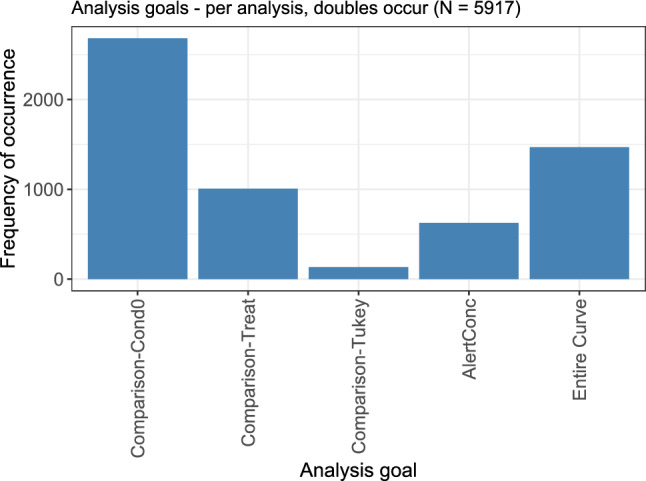
Frequency of occurrence for the different analysis goals. All goals that refer to ‘comparisons’ indicate some statistical testing

In this review, it was possible to assign more than one analysis goal to an analysis, such that for the 5670 analyses, in total, 5917 analysis goals were evaluated. The frequency distribution of the different goals is displayed in Fig. 8. All goals that refer to some kind of ‘comparison’ indicate that some statistical testing has been performed in the analysis. By far the most commonly observed analysis goal was the comparison against the lowest considered condition, which is often the control. This was followed by the consideration of the dose–response relationship in its entirety, without any explicitly tested comparisons, e.g., by describing a generally increasing or decreasing shape of the profile. The comparison between different dose–response relationships was the third most frequent goal, followed by the calculation of an alert concentration. Pairwise comparisons between all considered doses were only very rarely the goal of a dose–response analysis. Details on the used testing and modelling methods, and on the calculated alert concentrations, are given in the subsequent sections.

### Testing methods

**Fig. 9:**
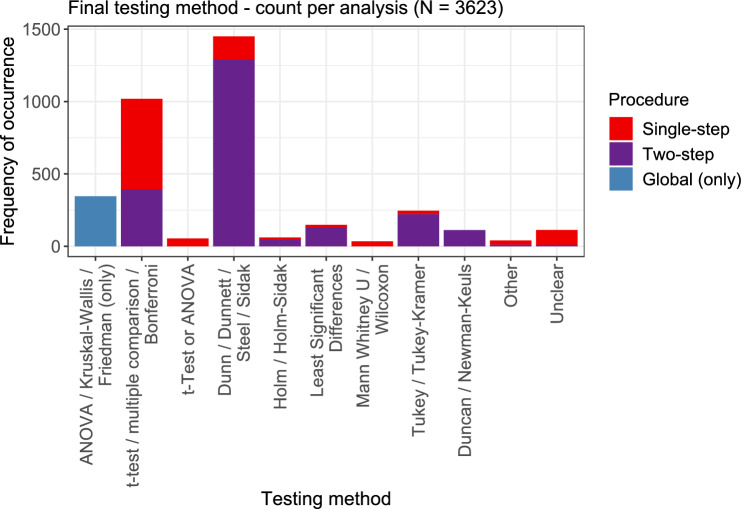
Overview of the different testing methods. Colors indicate the type of procedure, i.e., if only a global test was performed, or only multiple comparisons were performed (single-step), or the multiple comparison was performed as a post hoc test after some global procedure (two-step)

An overview of the testing methods used in the papers is given in Fig. 9. For 2217 of the displayed 3623 analyses, a two-step procedure was carried out, with an initial global test and a subsequent post hoc procedure. This is indicated by purple color in the bars. For 346 analyses, only a global test was performed, which does not allow statements about the individual doses.

The category Dunn/Dunnett/Stell/Sidak was most frequently observed, also in the majority of cases as part of a two-step procedure. These procedures mostly aim at simultaneously comparing the response values for each condition against the negative control. This is followed by the category *t*-test/multiple comparison/Bonferroni, which is a relatively broad category, since the multiple comparison method is not further specified. In this category, the two-step approach was chosen notably less often. All other categories occurred much less frequently. Among these, the Tukey/Tukey–Kramer method (i.e., considering all pairwise comparisons) is the one that was chosen most often, followed in that order by the method of least significant differences and the Duncan/Newman–Keuls test. The remaining methods were chosen only very seldom.

The still relatively large number of occurrences of the Duncan and the Newman–Keuls method is a bit surprising, since for these tests, it is known that the increase in power comes at the cost of not keeping the significance level. In the very popular software GraphPad Prism (see Section “Software”), the use of the Newman–Keuls test is explicitly discouraged,^1^ and the Duncan test is not even implemented due to its poor performance.^2^

While the two-step approaches are obviously very popular in published dose–response analyses, their usage is often discouraged based on statistical considerations. This is discussed in more detail in Section “Evaluation”.

### Modelling methods

**Fig. 10:**
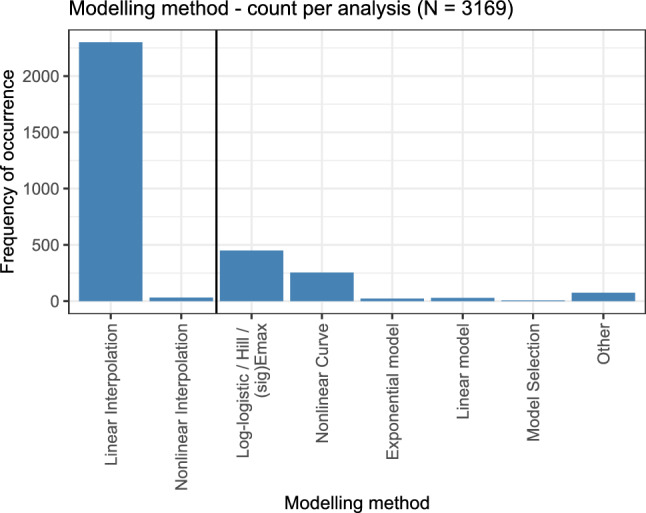
Number of times each modelling method was used, shown on a per-analysis level. The plot is divided by a line into the interpolation approaches (left) and the approaches with an underlying model function (right)

In contrast to testing methods as discussed above, modelling approaches allow to interpolate the dose–response relationship to arbitrary dose values. Figure 10 displays the number of times each modelling method was used. Here, a visual differentiation is made between interpolation-based approaches and approaches with an underlying model function. Linear interpolation was the by far most frequently used approach; see also Fig. 7. Among the not interpolated model approaches, the family of the log-logistic model (also called Hill or (sig)Emax model) was the most popular one. For a notable number of analyses, the specific model function could not be retrieved from the paper. Other models, or a model selection approach taking several models into account, were only used negligibly often.

A more detailed discussion on the advantages and challenges when using model fits to analyse dose–response data is given in Section “Evaluation”.

### Alert concentration

**Fig. 11:**
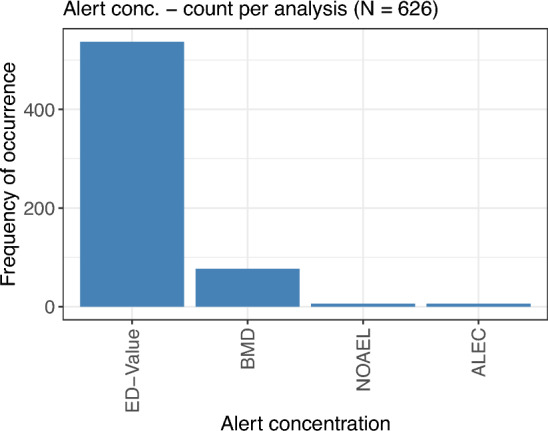
Number of times each alert concentration was calculated, all based on a modelled or interpolated dose–response curve

The calculation of an alert concentration is based on a curve describing the dose–response relationship. Figure 11 displays the frequency of the different alert concentrations, evaluated per individual analysis. ED values were the by far most commonly calculated alert concentrations, with a frequency of over 500 out of all 626 analyses for which an alert concentration was calculated. The second most considered alert concentrations was the BMD, while the occurrences of NOAEL and ALEC were negligible.

Technically, whenever a comparison against the negative control is performed (as often indicated by the stars for the barplots), a special case of the lowest observed effective concentration [LOEC, (Kappenberg et al. 2021)], where only statistical significance and no biological relevance is of interest, is calculated. Generally, the LOEC is defined as the lowest concentration where the difference in mean response values significantly exceeds some pre-defined threshold, so the multiple comparisons against the control in the default correspond to a threshold of 0. In this review, however, the emphasis with respect to alert concentrations was put on those values that were calculated with the explicitly formulated goal of finding a pre-specified alert concentration, such as ED values or BMD, and not on those analyses where just some comparison against the control was performed.

The typical usage of the NOAEL is problematic and also often criticized. The general definition of this alert concentration is that it is the highest condition for which no significant difference in comparison to the control can be observed. In the framework of statistical relevance testing, not being able to reject the null hypothesis does not necessarily correspond to the absence of an effect (Dorato and Engelhardt 2005; Delignette-Muller et al. 2011). To correctly assume the absence of an effect, the statistical framework of equivalence testing would be required, but, generally, the use of other measures such as the BMD—which, in addition, makes use of the entire data, while the NOAEL only considers the respective condition levels—should be preferred (Jensen et al. 2019; OECD 2014)

Most of the calculated alert concentrations were based on fitting a (parametric) curve. However, for a small number of dose–response relationships that are displayed by a linear interpolation, ED values were calculated as the concentration where the interpolated curve intersects with a pre-defined percentage value. These ED values thus only depend on the response values of two neighboring conditions and do not exploit the possibility of making use of the entire data for the fitting of a model. Such ED values should be interpreted with caution, as they are strongly influenced by random variation.

### Software

**Fig. 12:**
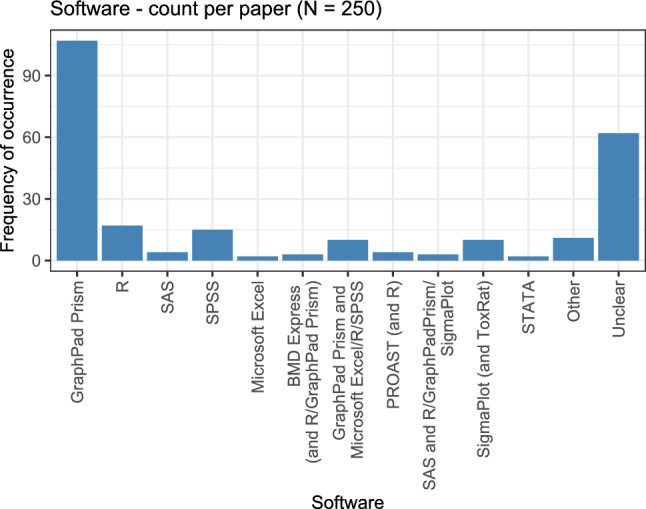
Overview over the different softwares that were used for analysing dose–response data and the respective frequencies of occurrence

The final variable of the univariate analysis is the used software program. This is considered purely on a per-paper basis, i.e., for 250 observations (papers). Figure 12 shows the softwares used for analysing dose–response data and the respective frequencies of their occurrence.

For more than 60 out of the 250 papers with dose–response analyses, the information about the software used could not be retrieved. For the remaining papers, GraphPad Prism (www.graphpad.com) was used in more than half of the cases, sometimes in combination with other programs. The statistical programming language R, IBM’s software for statistical data analysis SPSS, and the software for scientific graphing and data analysis SigmaPlot were used comparatively often. Other programs were mentioned comparatively rarely, and sometimes also in combinations of several softwares.

### Bivariate displays

In addition to the univariate analyses presented in the previous sections, interesting combinations of the variables are now considered in a bivariate way. Results are presented via mosaic plots. Not all possible combinations are shown, but five pairings are chosen based on the additional information they convey. The bivariate analyses are shown on a per-analysis level only (i.e., not on a per-paper level), since the plots for the univariate analyses show very similar results for both approaches.

**Fig. 13:**
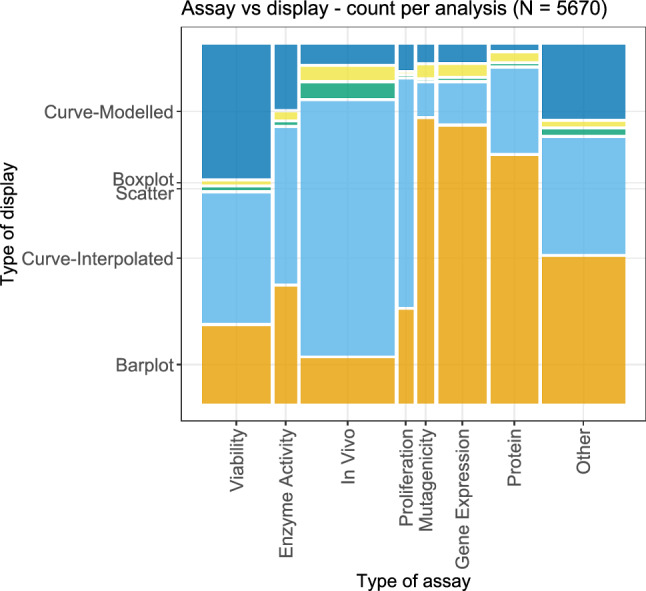
Bivariate plot of the type of assay and the type of display for each considered dose-response analysis

The first pairing, consisting of the type of assay and the type of display, is shown in Fig. 13. One observation here is that the display via ‘Curve-Modelled’, i.e., with an underlying (parametric) model, was used vastly more often for viability assays than for the other assays. The results from in vivo assays and proliferation assays were displayed via some interpolation in the majority of cases, and the proportion via interpolation was also highest for these types of assay, compared to other types. For mutagenicity, gene expression, and protein assays, barplots were used most often as a method of display. Overall, notable differences between the type of assays with respect to the types of displays can be seen.

**Fig. 14:**
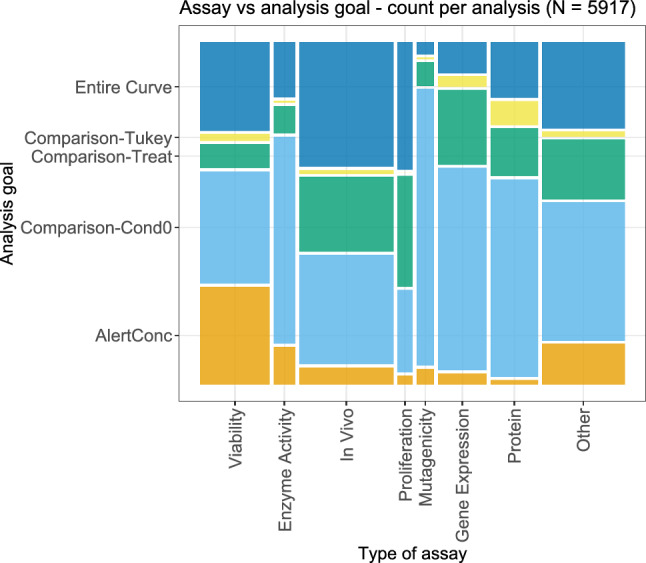
Bivariate plot of the type of assay and the corresponding analysis goal for each dose–response analysis. Some analyses had more than one goal; in such cases, they were counted multiple times in this plot

Figure 14 shows the bivariate relation between the type of assay and the corresponding analysis goal. Since, for some dose–response analyses, more than one analysis goal could be identified, the associated assays were duplicated correspondingly often. Across assay types, the analysis goal of calculating an alert concentration was most common for the viability assays, which in principle fits to the previous observation that modelling was performed more often for this type of assay. In vivo, proliferation, and also viability assays corresponded to the general goal of considering the (fitted) curve in its entirety comparatively often. The comparison against the lowest considered condition value was a very common goal especially for mutagenicity, gene expression, and protein assays.

**Fig. 15:**
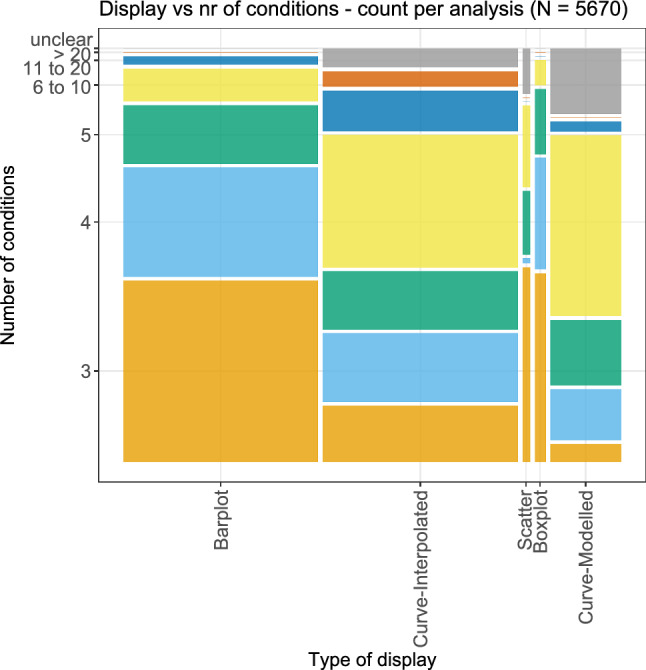
Bivariate plot of the different types of displays against the number of conditions. These numbers were further summarized in comparison to the Fig. 4

A bivariate plot of the type of display and the number of conditions is shown in Fig. 15. In comparison to the previous (univariate) plot in Fig. 4, the number of conditions was summarized in fewer categories, i.e., the numbers 6–10 form only one category, as do the numbers 11–20. It can clearly be seen that when the results were displayed via barplots, scatter plots, or boxplots, the number of conditions was generally smaller, with only three conditions being the largest category, respectively. For the display via some interpolation or even via the modelling of a curve, the number of conditions was very often higher. Especially, for the display via a modelled curve, only three conditions occurred very seldom, with the most frequent category being 6–10 conditions.

**Fig. 16:**
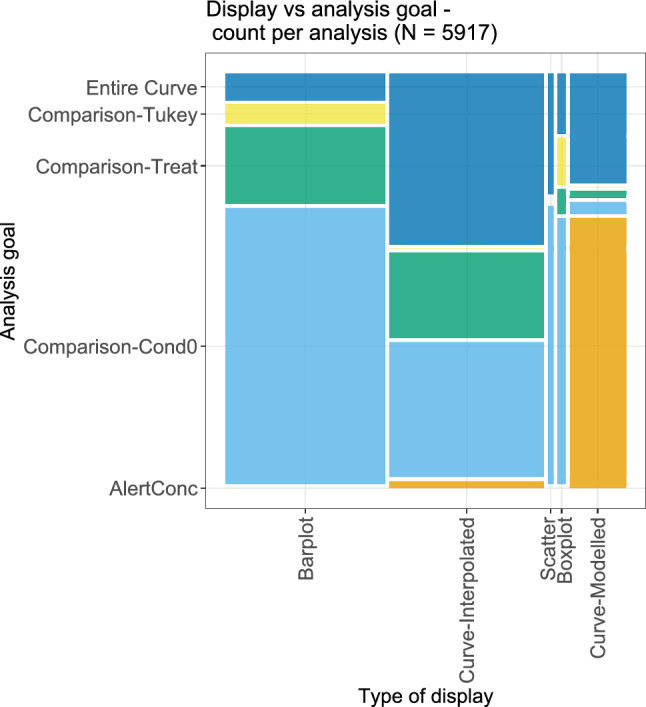
Bivariate plot of the different types of display and the corresponding analysis goal. Some analyses had more than one goal; in that case, they appear several times in this plot

The next bivariate analysis, shown in Fig. 16, considers the type of display and the analysis goal. As before, since more than one analysis goal was possible for each dose–response analysis, the associated type of display was duplicated correspondingly often. Overall, the results are in line with the previous findings. For the display of results via barplots, the most frequent corresponding analysis goal was the comparison against the lowest condition value. The calculation of alert concentrations was the most frequent analysis goal when displaying the results via a modelled curve. For other displays, alert concentrations were rarely the analysis goal, only in few cases for the display via a curve that was based on some interpolation. For modelled and for interpolated curves, often also the entire curve was the analysis goal of interest. Only for the display via barplots or boxplots, the consideration of all pairwise comparisons was a relatively often selected analysis goal.

In conclusion, differences between the types of assays and the corresponding choice of statistical methods, both for the display of the results and for the analysis goal, could be observed. The different types of displays were also associated with different types of analysis goals, and, possibly due to the different requirements for the respective statistical method, also corresponded to different numbers of measured conditions.

**Fig. 17:**
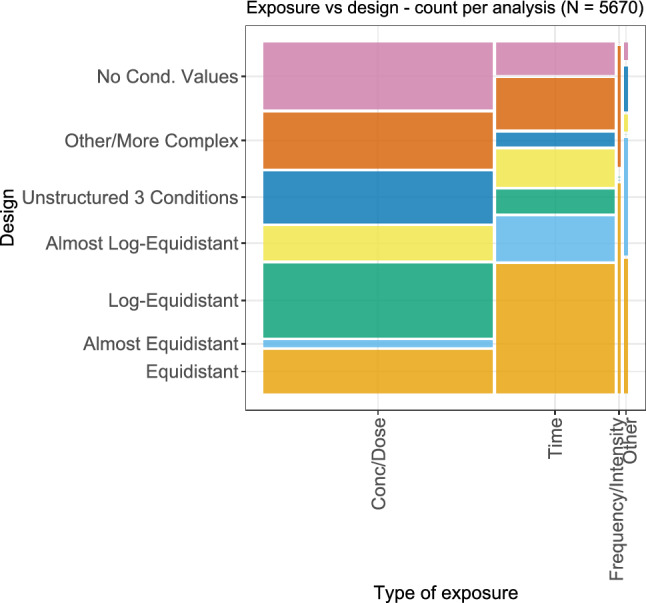
Bivariate plot of the different types of exposures and the corresponding chosen design for each considered dose–response analysis

While from a statistical point of view, in principle all types of condition values (concentrations, doses, times, etc.) are equivalent, the differences in their experimental handling can affect certain aspects of the statistical consideration, such as the design: From an experimental point of view, concentrations are often administered in dilution series, and thus on a multiplicative scale, while the measurement of time points can be arbitrary. The relationship between the type of exposure and the corresponding design is shown in Fig. 17. Here, it can be seen that equidistant or almost equidistant designs were far more common for time–response relationships than for concentration– or dose–response relationships, where log-equidistant and almost log-equidistant designs (i.e., designs on a multiplicative scale) were observed more often. In addition, the case that no condition values could be retrieved from the respective plot is present more often for concentration and dose as exposure. Since missing condition values were often due to the display of the analyses on a logarithmic axis, this could also hint at in principle more designs on a multiplicative scale. For the very few cases of a frequency or intensity as exposure, the designs were either equidistant, or in the category other/more complex.

### Evaluation

In this section, three aspects of the analysis of dose–response relationships are discussed in more detail, particularly with respect to statistical properties. These three aspects are the choice of statistical design, the use of two-step procedures in a multiple testing context, and the advantages of modelling dose–response relationships.

The first aspect considers the advantages of deciding on a suitable design based on statistical considerations. As shown in Fig. 17, the chosen design is dependent on the type of the exposure within the experiment. Nevertheless, from the statistical point of view, the type of exposure is less relevant for the design of the experiment than its actual analysis goal.

If a multiple testing for the comparison of effects at different conditions is planned, it is appropriate to fix the conditions to the values of interest. Moreover, the number of observations at each of these conditions should be equal to maximize the power of the later used multiple testing procedure, as shown in Wu and Hamada (2011). However, the performance of multiple testing methods, especially ANOVA, decreases if the number of different conditions of interest increases beyond five (Bornkamp et al. 2007). As a consequence, the modelling of the condition–response relationship should be preferred, as soon as the effects of more than five different conditions are of interest (further advantages of modelling are discussed below).

If modelling is planned, the usage of the frequently occurring equidistant and log-equidistant designs (cf. Fig. 5) might be inappropriate: These designs might contain conditions whose observations do not describe the relevant part of the parametric model and will, therefore, result in a bad fit. Instead model-specific optimal design strategies (like the usage of *D*-optimality or compound optimality criteria) result in allocations of conditions which optimize the quality of the resulting parametric model in terms of variance substantially (Pinheiro and Bornkamp 2017).

The second important point is about the use of two-step procedures when applying pairwise tests or simultaneous tests against a negative control. As can be seen in Fig. 9, applying first a global test (e.g., ANOVA) and then some so-called ‘post hoc’ test is a popular approach. This procedure is also required by some of the softwares used for analysing data, and it is the convention in certain fields of research. However, this might lead to results that are too conservative, meaning that group-wise differences might exist that are not identified via this procedure, in cases when the global test does not reject the global null hypothesis, and pairwise comparisons would only be considered after a rejection of the global null hypothesis (Hothorn 2016; Midway et al. 2020). The choice of appropriate models for the multiple comparisons themselves is another challenge, where Midway et al. (2020) gives some practical guidance.

The third and final aspect to be discussed in more detail refers to the advantages of modelling dose–response relationships by some underlying (parametric) model instead of considering only the actually measured condition values. Especially, with respect to the calculation of alert concentrations or the analysis of the overall shape of a curve, fitting a model to the data points has the advantage that it makes use of all data points simultaneously. In contrast, for interpolation approaches and for pairwise testing, typically only two conditions are used, respectively. The precise determination of an alert concentration from an interpolated curve depends on only two neighboring data points.

However, a major challenge in modelling is the choice of an appropriate model function. Some applications allow the selection of a model based on previous knowledge about typical dose–response relationships, such as a general sigmoidal shape (captured by the family of log-logistic functions) for viability assays (Krebs et al. 2020, e.g.). For other applications, the model selection needs to be performed in a data-driven way. A direct approach would be to compare several potential models in terms of some information criterion, or to use a two-step procedures such as the multiple comparison and modelling (MCP-Mod) approach (Bretz et al. 2005). This approach has also been applied to gene expression data and it has shown reasonable results (Duda et al. 2022).

While analysing viability assays with modelled dose–response curves and, if applicable, calculating ED values based on these curves is already quite common (see Fig. 13), recent research also considers gene expression data and the possibility to calculate the alert concentration ‘LEC’ (lowest effective concentration), which is a model-based alternative to the popular ‘LOEC’ (Kappenberg et al. 2021; Möllenhoff et al. 2022). One advantage of model-based alerts is that they do not depend on the design of the experiments; in that, they can take any value in between actually considered condition values.

## Guidance for statistical design and analysis

The results from Chapter 3 show that various aspects of dose–response experiments must be evaluated from a statistical point of view. The three phases planning, executing, and analysing a dose-response experiment each include several steps that should be considered as an entire analysis process with strong relations between the different steps. As a specific example, in the two-step procedure ‘MCP-Mod’ (Bretz et al. 2005), parametric modelling of dose-response curves comprises two steps: the application of multiple comparison procedures against the negative control (‘MCP’) and the final fitting of a curve (‘Mod’).

In the next section, a specific guidance strategy for planning, executing and analysing a dose–response experiment from a statistical point of view is proposed. In Section “Available software”, some software tools for facilitating the individual steps are suggested.

### Guidance: DENMAR

The first step of a toxicological dose–response experiment, after deciding on the biological assay and the type of exposure, is the **design**. This concerns both the choice of the condition values and the respective allocation of the sample sizes. While a lot of these considerations are based on available biological knowledge, the respective analysis goal of the experiment must also be considered when deciding on an optimal design. After that, the actual **experiment** can be conducted and data are obtained.

Depending on the type of experiment, the next step consists of the pre-processing and the **normalization** of the data. Examples for pre-processing are, e.g., for gene expression data, given by the robust multiarray average (RMA) algorithm (Irizarry et al. 2003), or for some viability assays by translating fluorescence intensities to numbers, or by removing background noise by some standardized procedures. The normalization then requires additional steps, such as converting numbers to percentages or removing possible batch effects by taking the difference of condition values and control values. However, the specific choice of pre-processing and normalization steps should always be tailored to the type of assay and the statistical approach of analysing it, since in some cases [e.g., when analysing RNA-Seq data with the DESeq2 approach (Love et al. 2014)], pre-processed, but unnormalized data are required.

In the actual statistical analysis, the dose–response data are analysed as a whole, which can be considered as the **modelling** of the relationship between dose and response. Results from the literature review show that currently most emphasis is still on comparing the actually measured conditions with the negative control or on performing linear interpolation for easier visualization of the data. Specifically, the fitting of a (parametric) model to the data to better capture the dose–response relationship should be considered, due to the advantages discussed in Section “Results of the literature review”. Here, it is important to think about suitable models in advance, based on prior biological or statistical knowledge, or to take model selection or model averaging approaches into account.

As a frequent goal of toxicological research, the calculation of an **alert concentration** is of interest. This could both mean to analyse the results of multiple comparison procedures against a negative control (i.e., Dunnett-type tests, ‘LOEC’), or to actually calculate a specific alert concentration (ED value, BMD, ALEC, ...) based on a fitted model.

The final and very important step is to **report** all results in a way that the analysis could be completely reproduced. This reporting should include all relevant information about the experimental setup (choice of design, sample sizes), the execution of the experiment (often by referring to some standard operating procedures), the pre-processing and normalization, and the choice of the analysis method (the specific testing method used, or the specific type of model used, and the type of alert concentration). In the literature, often not all of this information could be retrieved from the published dose–response analyses, but it is crucial to keep completeness and reproducibility in mind when reporting results of dose–response experiments.

The previously discussed steps are summarized in Table 2, which proposes the six steps as a minimal requirement for an analysis pipeline via the newly introduced ‘DENMAR’ (design, experiment, normalize, modelling, alert concentration, report) approach.

**Table 2:** Summarized steps for planning, executing, and analysing a dose–response analysis

**D**	Design	Plan the design of the experiment, considering the analysis plan
**E**	Experiment	Conduct the toxicological experiment
**N**	Normalize	Perform normalization, tailored to the type of assay (e.g., remove batch effects, convert to percentages, etc.)
**M**	Modelling	Model the dose–response relationship; if possible consider fitting a parametric model
**A**	Alert concentration	Calculate the alert concentration of interest (e.g., ED values, NOAEL, BMD, LOEC (Dunnett-type test))
**R**	Report	Report precisely all applied methods (testing/modelling) and the resulting conclusions

### Available software

In addition to the previously discussed software solutions for analysing dose–response data (see Section “Software”), some specific software solutions are pointed out here that help with certain aspects of the recommended steps for planning, executing, and analysing a dose–response analysis.

The R shiny app DoseResponseDesigns (https://biostatistics.dkfz.de/DoseResponseDesigns/, Holland-Letz and Kopp-Schneider (2021)) allows the calculation of optimal designs when assuming underlying log-logistic or Weibull functions, as well as comparing a specific design to the respective optimal design. This also extends to combination experiments with two compounds.

Two software solutions for fitting parametric dose–response curves and for calculating estimates for the BMD alerts based on these fits are given by BMCeasy (http://invitrotox.uni-konstanz.de/BMCeasy/, Krebs et al. (2020)), which is specifically aimed at viability data, and BMDExpress2 (https://www.sciome.com/bmdexpress/, Phillips et al. (2018)), which is aimed at analysing genomic data.

## Discussion and conclusion

There is a discrepancy between the state of the art in statistical methodological research and the methods used for analysing dose–response experiments in toxicological research. In this paper, the extent of this discrepancy is quantified via a comprehensive literature review in all publications of the year 2021 from the three major toxicological journals ‘Archives of Toxicology’, ‘Cell Biology and Toxicology’, and ‘Toxicological Sciences’. This review addressed various aspects of dose–response analyses in terms of underlying biological considerations, statistical design considerations, and statistical analysis considerations.

Three major results are discussed in detail. The first result is that there is a lack in using statistical design theory for the determination of the dose values and the respective allocation of sample sizes. The second aspect considers the testing procedure in pairwise comparisons, or multiple comparisons against a negative control. It was found that a global test often preceded the actual individual comparisons of interest, which can lead to an increase of false-negative results. The third aspect is that often only the actually measured concentrations were considered for further analyses or only linear interpolation was conducted. Instead, the choice of an underlying (parametric) model is beneficial in many applications, and allows interpolation between the actually measured doses. Based on the overall results of the literature review, guidance for planning, executing, and analysing a dose–response experiment is proposed. The steps are abbreviated as ‘DENMAR’, which stands for the aspects design, experiment, normalize, modelling, alert concentration, and report.

The two-step testing approach, where individual comparisons are only tested after obtaining a significant global test result, is still often encouraged by several software solutions for analysing dose–response data. In the review conducted here, it was not evaluated how often statistical analyses were stopped after non-significant results of a global test, i.e., how often a global test, e.g., ANOVA, was performed and the result was not significant, and therefore, no post hoc test was conducted afterwards.

For many specific toxicological assays conducted in pre-clinical research, the Organisation for Economic Co-operation and Development (OECD) has issued several guidelines, e.g., for the Ames assay (OECD 2020), for the Comet assay (OECD 2016) or, more general, for carcinogenicity studies (OECD 2018a), chronic toxicity studies (OECD 2018b), or the combination of both (OECD 2018c).

In a current guideline published by the European Food Safety Authority (EFSA), the BMD approach to calculating alert concentrations is clearly preferred over the NOAEL (EFSA Scientific Committee et al. 2022). Further, the recommendation is to perform model averaging of the BMD values that are based on the Bayesian fitting of a dose–response model.

In this work, however, the focus was not on such guidelines, but on general observations about the current practices in published toxicological research. For more details about the current state of the art in published toxicological research in connection with available guidelines, the reader is referred to Hothorn (2014).

In particular, also statistical researchers must be aware that only providing theoretical results about the best practices in designing and analysing dose–response data is not enough. The methodological research needs to be made available to practitioners via user-friendly software solutions. One way to achieve this are graphical user interfaces, as provided by the shiny package in R, which allows an easy creation of apps that can be hosted via websites (Chang et al. 2022). A very important aspect for software solutions also is to guide the user with respect to the correct reporting, such that also in more complex methodological situations, it is clear which method was used by the respective program. This ultimately enables reporting of methods and results in a reproducible way.

## Data Availability

Since no experimental data were collected, such a statement is not really applicable.

## Footnotes

1 https://www.graphpad.com/guides/prism/latest/statistics/stat_newman-keuls_method.htm, last accessed on 22.5.2023.

2 https://www.graphpad.com/support/faq/does-graphpad-prism-or-graphpad-instat-offer-duncans-multiple-comparison-test/ last accessed on 22.5.2023.
